# Take me where I want to go: Institutional prestige, advisor sponsorship, and academic career placement preferences

**DOI:** 10.1371/journal.pone.0176977

**Published:** 2017-05-11

**Authors:** Diogo L. Pinheiro, Julia Melkers, Sunni Newton

**Affiliations:** 1 Department of Sociology, Savannah State University, Savannah, GA, United States of America; 2 School of Public Policy, Georgia Institute of Technology, Atlanta, GA, United States of America; 3 Center for Education Integrating Science, Mathematics and Computing (CEISMC), Georgia Institute of Technology, Atlanta, GA, United States of America; Iowa State University, UNITED STATES

## Abstract

Placement in prestigious research institutions for STEM (science, technology, engineering, and mathematics) PhD recipients is generally considered to be optimal. Yet some doctoral recipients are not interested in intensive research careers and instead seek alternative careers, outside but also within academe (for example teaching positions in Liberal Arts Schools). Recent attention to non-academic pathways has expanded our understanding of alternative PhD careers. However, career preferences and placements are also nuanced along the academic pathway. Existing research on academic careers (mostly research-centric) has found that certain factors have a significant impact on the prestige of both the institutional placement and the salary of PhD recipients. We understand less, however, about the functioning of career preferences and related placements outside of the top academic research institutions. Our work builds on prior studies of academic career placement to explore the impact that prestige of PhD-granting institution, advisor involvement, and cultural capital have on the extent to which STEM PhDs are placed in their preferred academic institution types. What determines whether an individual with a preference for research oriented institutions works at a Research Extensive university? Or whether an individual with a preference for teaching works at a Liberal Arts college? Using survey data from a nationally representative sample of faculty in biology, biochemistry, civil engineering and mathematics at four different Carnegie Classified institution types (Research Extensive, Research Intensive, Master’s I & II, and Liberal Arts Colleges), we examine the relative weight of different individual and institutional characteristics on institutional type placement. We find that doctoral institutional prestige plays a significant role in matching individuals with their preferred institutional type, but that advisor involvement only has an impact on those with a preference for research oriented institutions. Gender effects are also observed, particularly in the role of the advisor in affecting preferred career placement.

## Introduction

The traditional pathways for PhD scientists and engineers have expanded considerably, and accordingly, individual’s career preferences have become more varied. Recent attention has been paid to the preparation and support of PhD recipients in STEM (science, technology, engineering, and mathematics) for non-academic careers, given growing interests and opportunities in government and industry, coupled with evidence of a growing PhD workforce without a corresponding growth in academic jobs [1–3]. Yet while there has been a growing body of research addressing non-academic pathways, there has been less attention paid to the nuanced career interests and opportunities within academe. Most notably, not all PhD scientists with aspirations for an academic career are interested in intensive *research* careers; some STEM PhD recipients prefer teaching focused careers, and such preferences are driven by a variety of personal and professional factors [2,4–10]. In considering these varied career path options, a question of interest is as follows: what factors determine an individual’s ability to realize their career goals within the academic career path, particularly with respect to obtaining a more research or teaching centric academic position? Does preference alone determine career placement?

Studies of academic job placement would suggest otherwise. First and foremost, multiple studies have found that departmental or institutional prestige of PhD-granting institution is a better predictor of job placement post-PhD than any individual measures [5,11–18]. Complicating this job search and placement process is the support of advisors, or lack thereof, in advisees’ pursuit of their preferred career paths. Evidence suggests that the extent to which an advisor encourages and assists an advisee in the job search process for non-faculty research positions varies considerably, often on the basis of the type of career the advisee seeks [1,3].

The aim of our research is to examine how individual academic career placement is affected by individual preference, doctoral institution prestige, and advisor support. Further, how do these factors work when a STEM PhD recipient pursues a path other than toward the most prestigious/competitive institutions? For all the attention that factors related to departmental prestige and advisors have received, the bulk of this attention has been concentrated on a relatively small set (~150) of the top research institutions. Yet the academic workforce is actually employed in more than 4,500 post-secondary universities and colleges nationwide [19]. To effectively investigate academic career preferences, researchers must look beyond the relatively narrow band of highly prestigious and research-centric career paths in the top research institutions. Our work is based on a large, National Science Foundation funded research study of STEM faculty members in four disciplines from 487 post-secondary institutions, including a range of teaching versus research-centric institutions at varying levels of prestige, in the United States. The results of our analysis show important effects of preferences, prestige and advisor support in academic placement, including important gender effects.

### Preferences and choice in the STEM academic job market

Employment outcomes in any labor market are dependent on two key factors: opportunity and choice, where *opportunity* refers to the jobs that are made available by employers, and *choice* entails how the workers select from among those opportunities[20]. There has been considerable work addressing the *opportunity* aspect of the academic labor market, with studies estimating the impacts of ascriptive (e.g., gender, race, nationality, institutional prestige) or achieved (e.g., publications, grants, awards) characteristics on career placement and outcomes. Previously investigated academic career outcomes include institutional prestige [11,13], salary [21], early career productivity[22], and rank/advancement [23].

Attention to career preferences, or *choice*, has been more recently motivated by a challenging and restricted PhD job market [24], coupled with expanding interest of STEM doctoral recipients in non-research careers. A growing body of work, as well as the development of policy initiatives, addressing these preferences has mostly focused on non-academic careers [3,25–28]. However, comparably less attention has been paid to career choices within academe, including interests in particular types of institutions and preferences for careers with a teaching vs. a research focus. The academic workplace is broad and marked by a significant contrast in expectations and focus of faculty work between research and teaching institutions, large and small colleges and universities, and liberal arts colleges. Evidence from myriad studies indicates that work-family balance, individual’s undergraduate experience, teaching interests, spousal-employment constraints, dual academic couple career challenges, and/or a desire to “give back” to the community shape preferences for academic positions outside of the most prestigious and research intensive institutions [2,4–10]. These various considerations driving individual’s career preferences, combined with the increasingly varied academic career options, present a potentially fruitful area of inquiry into *opportunity* and *choice* in the academic labor market.

Much of what has been learned about shifting career preferences in the contemporary academic labor market has focused on doctoral students as they refine their aspirations and enter the active job market. Observations of “branching” of interests into varied career paths is increasingly the norm, particularly in some STEM disciplines [25], and shifts in career preferences for or against academia have been observed during PhD pursuit [29]. Individual demographic characteristics have also been linked to academic career preferences, changes in these preferences, and factors driving these preferences and changes in them, over the course of graduate students’ progressions through graduate school[30–34]. This is important for many reasons, including the fact that women and underrepresented minorities in STEM disciplines are disproportionally represented in non-doctoral-serving institutions [35], and women are represented at higher rates at teaching-focused as compared to research-focused schools [36].

### Placement, preferences and prestige

Existing research on academic prestige indeed finds that the prestige of PhD-granting institution is the main force shaping the academic labor market [5,11,18,37,38]. Long, Allison and McGinnis [5], for example, tracked over 200 male PhDs in biochemistry and reported that prestige of PhD granting institution had a significant and substantial effect on prestige of the institutions where candidates were subsequently employed. More importantly, their research also found that these effects were independent of any pre-employment productivity, and that pre-employment productivity had no significant impact on a candidate’s position within the “prestige hierarchy.” Similar results have been found for faculty hires in mathematics, chemistry, biology, physics, sociology, and several other disciplines [12,15,39,40]. Recent work in the field of sociology [13] showed that the accumulation of resources and opportunities coupled with prestige has market advantages, and provides evidence that institutional prestige is especially important in determining employment opportunities at more prestigious schools, net of key individual characteristics. Here, symbolic capital (i.e., the prestige of institutions within the field of academia) then plays a role in the development of prestige hierarchies.

If we consider academic prestige as a type of capital, partly symbolic and partly social [41], we might expect that individuals with a teaching preference from more prestigious departments may be more likely to work at teaching-focus institutions, and individuals with a research preference from prestigious departments may be more likely to work at research-focused institutions. If so, this may mean that the effect of prestige might be even more significant than previously thought, given that faculty with their degrees from prestigious doctoral departments but who hold less prestigious positions may have followed such a path largely due to their personal preference rather than limited employment options. Prestige may interact with career path preferences, where for example, individuals from more prestigious departments with a teaching preference may be more likely to work at Liberal Arts colleges (prestigious teaching institutions) than any other type of institution. And, individuals from prestigious departments with a research preference may be more likely to work at Research Extensive institutions (which are the most prestigious research-centric) than at any other type of institution. Given this expectation, we hypothesize:

Hypothesis 1: Individuals from more prestigious departments are more likely to work in institutional types that best match their (research or teaching) preferences.

### Advisors and career trajectories

The relationship between a graduate student and his/her advisor plays a prominent role in shaping the students’ graduate school experience. In fact, some scholars argue that this relationship is the single largest determining factor in a graduate student’s overall experience and persistence in a PhD program [42–44]. Observing how the role of one’s PhD advisor shapes an individual’s perceptions of a certain career path, and may impact their career preferences [45]. Further, student-reported quality of relationship with advisor has been shown to be a significant predictor of reported preference for a faculty position at a research university [1,46].

The academic workplace has been frequently described as being shaped by “sponsored mobility” [15,47,48], where “mobility is like entry into a private club where each candidate must be ‘sponsored’ by one or more of the members”[48]. Advisors and other faculty act as these “sponsors”, opening doors, or failing to do so, for different types of opportunities. Doctoral advisor visibility [15], productivity [49], and co-authorships with their students[22,50] have each been found to have significant and frequently long-lasting effects on academic careers. These findings generally point to a dual process through which advisors affect scientific careers. On one hand, there is the ascriptive aspect of sponsorship, where advisor visibility raises the profile of individual candidates, thus increasing their success on the academic market. On the other hand, having a more involved and productive advisor has been shown to facilitate socialization into academic careers. For example, Fuerstman and Lavertu [51] have found that search committees consistently rank recommendation letters as one of the most important factors influencing hiring decisions, and Ladner, Bolyard, Apul and Whelton [52] stressed the importance of direct advice on application and negotiation strategies for academic engineers.

While advisor relationships may shape career preferences, our interest is focused on the tangible engagement of the advisor in the job search process itself. Our expectation here is that advisor involvement in a candidate’s job search process will lead to a better matching of candidate’s preferences and employment. Advisor involvement should result in better market outcomes, which in turn should provide individuals with more opportunities to choose a suitable institution that matches their preferences. With this in mind, we propose the following hypothesis:

Hypothesis 2: Individuals who were more actively sponsored by their advisors are more likely to have a job at an institution that matches their preferences with regards to teaching or research orientations.

**Fig 1:**
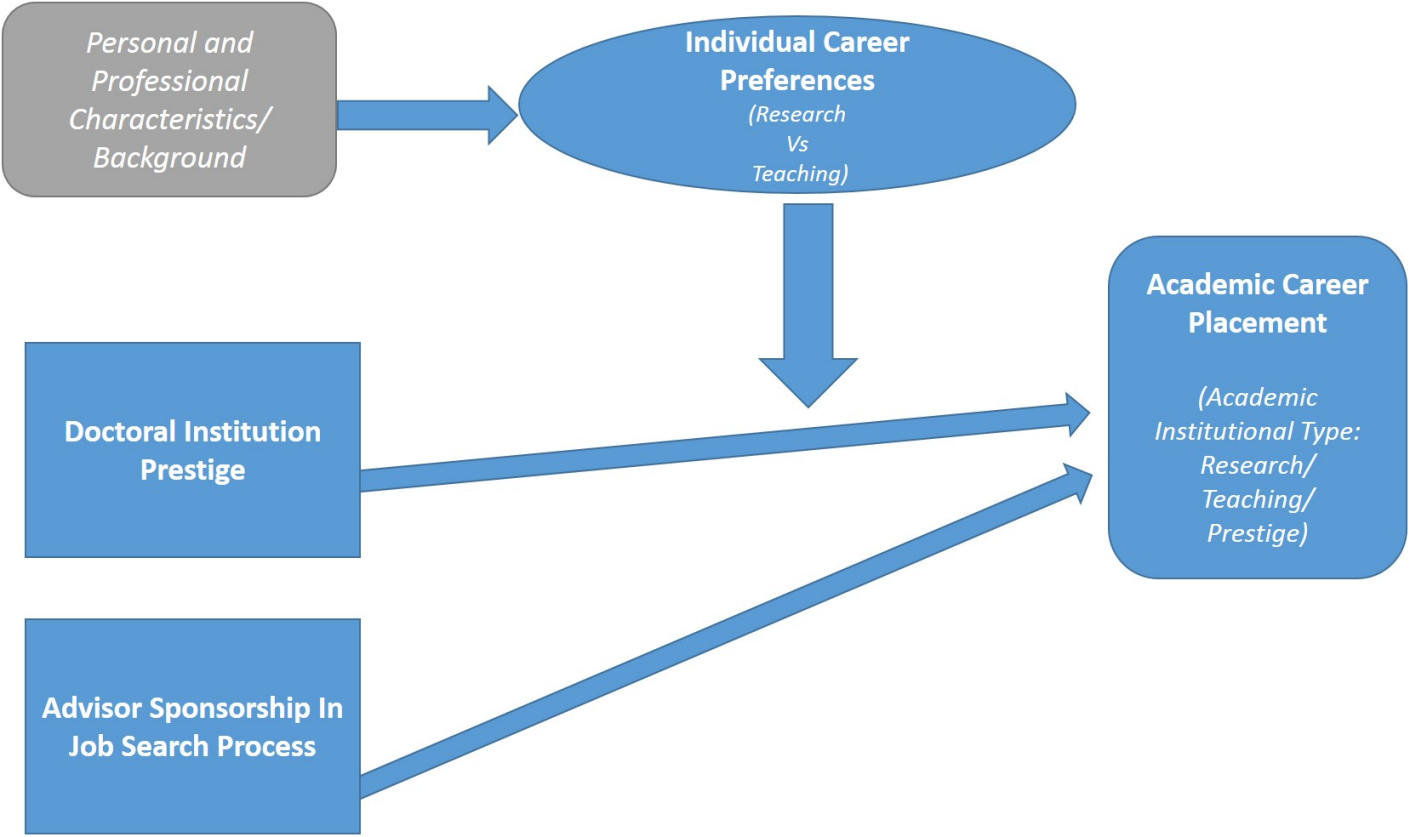
Summary research model.

Our overall model, depicted in Fig 1, illustrates these expected relationships and factors that explain institutional placement, together with a number of disciplinary, demographic and other factors that are relevant to these outcomes.

## Results

The purpose of this research was to examine how institutional prestige, coupled with individual placement preferences and other factors relevant to the search process, matters for academic job placement type. To contextualize our analysis, we first present a descriptive summary of the initial academic career preferences and the extent of job mismatch in our data. We then present the statistical models used to test the hypotheses specified above. Details on methods and these models are at the end of this article.

### Descriptive analysis: Academic career placement preferences

**Fig 2:**
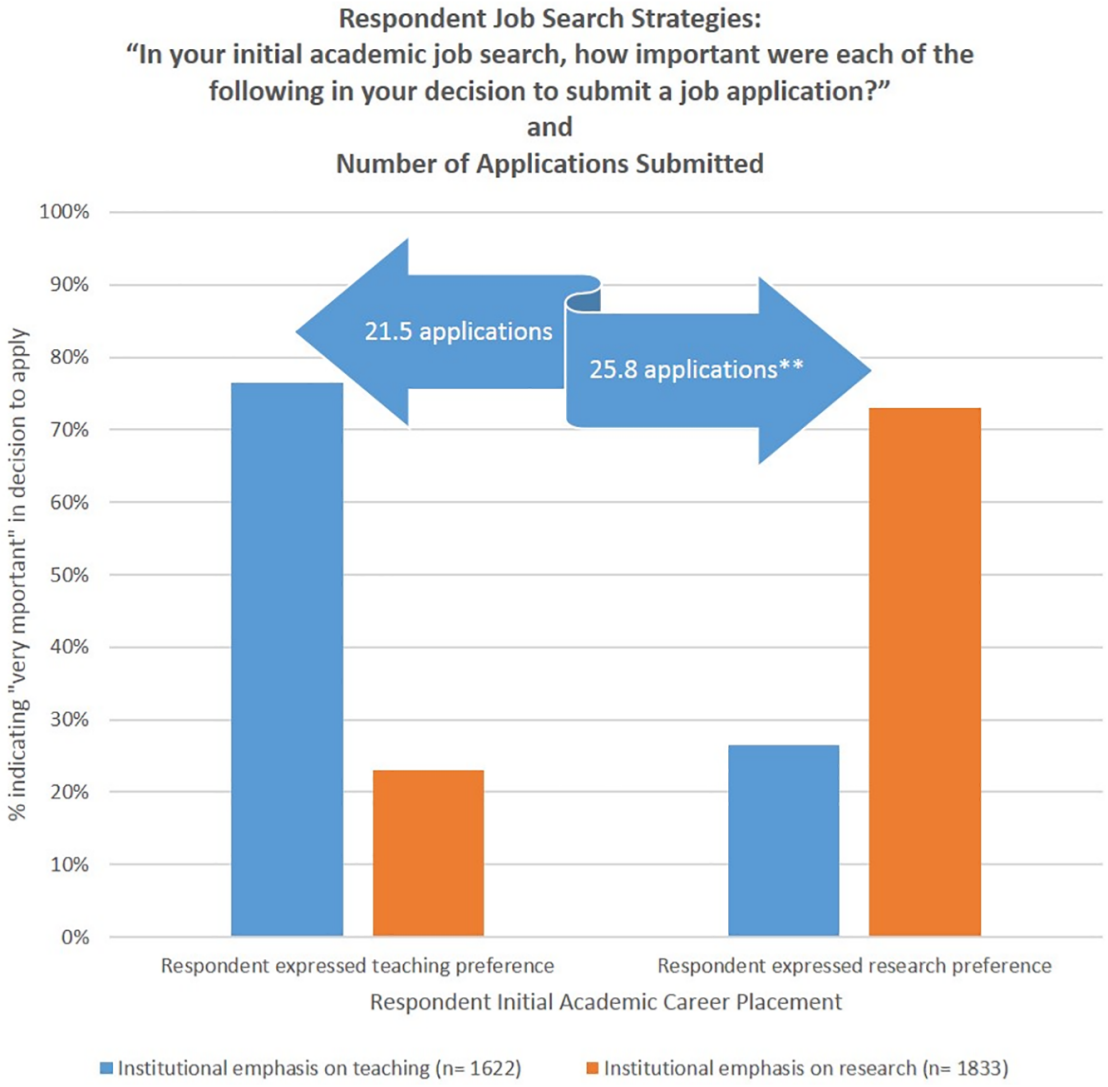
Job search preferences and application strategy.

When asked about their top choice for their first post-PhD career placement, over half of respondents (52%) indicated a research intensive environment, with fewer (34%) preferring a teaching intensive position. Men were more heavily represented in the group of respondents who preferred a research intensive environment (62% male and 38% female), while respondents who preferred a teaching intensive environment were almost evenly split by gender (51% men and 49% for women). As shown in Fig 2, job search strategies generally reflected these preferences, with about 75% of respondents in either preference group primarily targeting institutions with that emphasis. However, there were differences in the number of applications submitted, with those with a primary research interest submitting statistically significantly more job applications (25.8 versus 21.5) than did those with a teaching preference. Further, these individuals may be casting a broader net in the job search process, while those with a teaching preference may be focusing more tightly on institutions consistent with their interests. Our results show that individuals with a research preference applied at a slightly higher rate to teaching intensive institutions, while those with a teaching preferences did not show the same pattern in applying to research-intensive schools.

### Mismatch and academic career placement preferences

**Fig 3:**
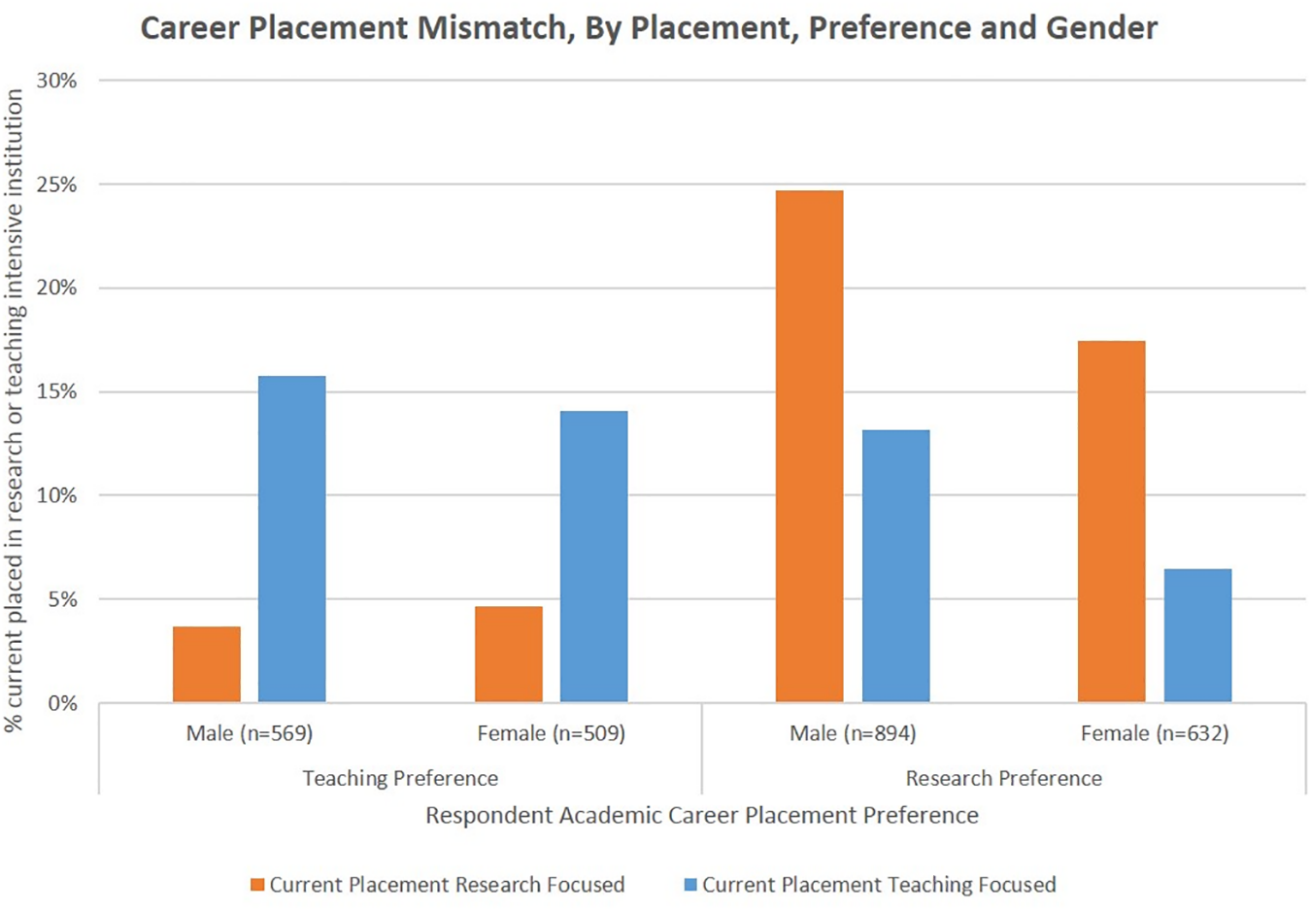
Career mismatch, by preference and gender.

To what extent do faculty experience a career placement mismatch, landing in an academic position that is not in line with their teaching or research preferences? To address this, we examine anyone who had a teaching preference and is currently employed at a research institution and vice versa. As shown in Fig 3, while only about one-third of respondents appear to have a mismatch in career placement, there are interesting differences in terms of the *type of mismatch*. The descriptive results show that men are substantially more likely to report being mismatched by having a research preference and working at a teaching oriented institution, while women report similar rates of mismatch across preferences.

What factors explain this mismatch? As shown in Table 1, only a few variables seem to consistently predict mismatches. Respondents with doctorates from prestigious institutions are about one-third (0.751) less likely to be mismatched, and dissertation award winners are more than 50% less likely to be mismatched (0.439). Both are expected and suggest the influence of recognition in the career placement process.

**Table 1:** Logistic regression: Factors associated with academic career placement mismatch, odds ratios.

	Full Sample			Male Faculty			Female Faculty		
	Odds ratio	Sig	SE	Odds ratio	Sig	SE	Odds ratio	Sig	SE
***Career Preference/Support***									
***Teaching preference***	2.243	***	0.326	2.23	***	0.447	2.172	***	0.363
***Advisor Sponsorship***	0.909		0.074	0.905		0.095	0.925		0.101
Teaching preference* advisor sponsorship	1.036		0.138	1.202		0.225	0.799		0.122
Teaching preference* doctoral prestige	1.282	*	0.18	1.328		0.257	1.189		0.204
***Doctoral Training Background***									
***Doctoral Institution Prestige***	0.751	***	0.063	0.758	***	0.08	0.756	**	0.089
***Dissertation Award***	0.439	***	0.13	0.468	**	0.159	0.281	***	0.134
***Year of PhD***	1.001		0.006	1.003		0.007	0.997		0.008
***Discipline***									
***Biochemistry***	0.572	***	0.095	0.458	***	0.101	0.851		0.202
***Civil Engineering***	0.919		0.167	0.814		0.189	1.307		0.325
***Mathematics***	0.97		0.156	0.918		0.199	1.068		0.212
***Demographics***									
***Dependent Child at PhD Completion***	1.024		0.152	1.043		0.195	0.984		0.22
***Female***	1.026		0.132						
***First Generation College Graduate***	1.106		0.156	1.135		0.204	1.082		0.222
***African American***	1.182		0.307	1.502		0.541	0.651		0.24
***Hispanic***	0.885		0.247	0.894		0.343	0.891		0.357
***Native American***	0.886		0.612	1.084		0.829	0.35		0.397
***Asian***	0.992		0.142	1.015		0.186	0.995		0.219
***Other Race/Ethnicity***	4.646	**	2.863	5.171	**	4.335	3.554		2.999
***Constant***	0.037		0.445	0.001		0.009	210.2		3,561
***N***	2,555			1,418			1,137		
***Log Likelihood***	-3071			-2126			-928.9		
***Pseudo R2***	0.053			0.057			0.056		

*** p<0.01

** p<0.05

* p<0.1

Faculty with a teaching preference, however, are also more likely to be mismatched than those with a research preference, even when coming from prestigious institutions. Further, disciplinary effects are also observed–male biochemistry faculty are less likely to be mismatched, although results for women in biochemistry are not significant. Notably, there were no differences in terms of gender or level of advisor sponsorship in relation to likelihood of being mismatched.

**Fig 4:**
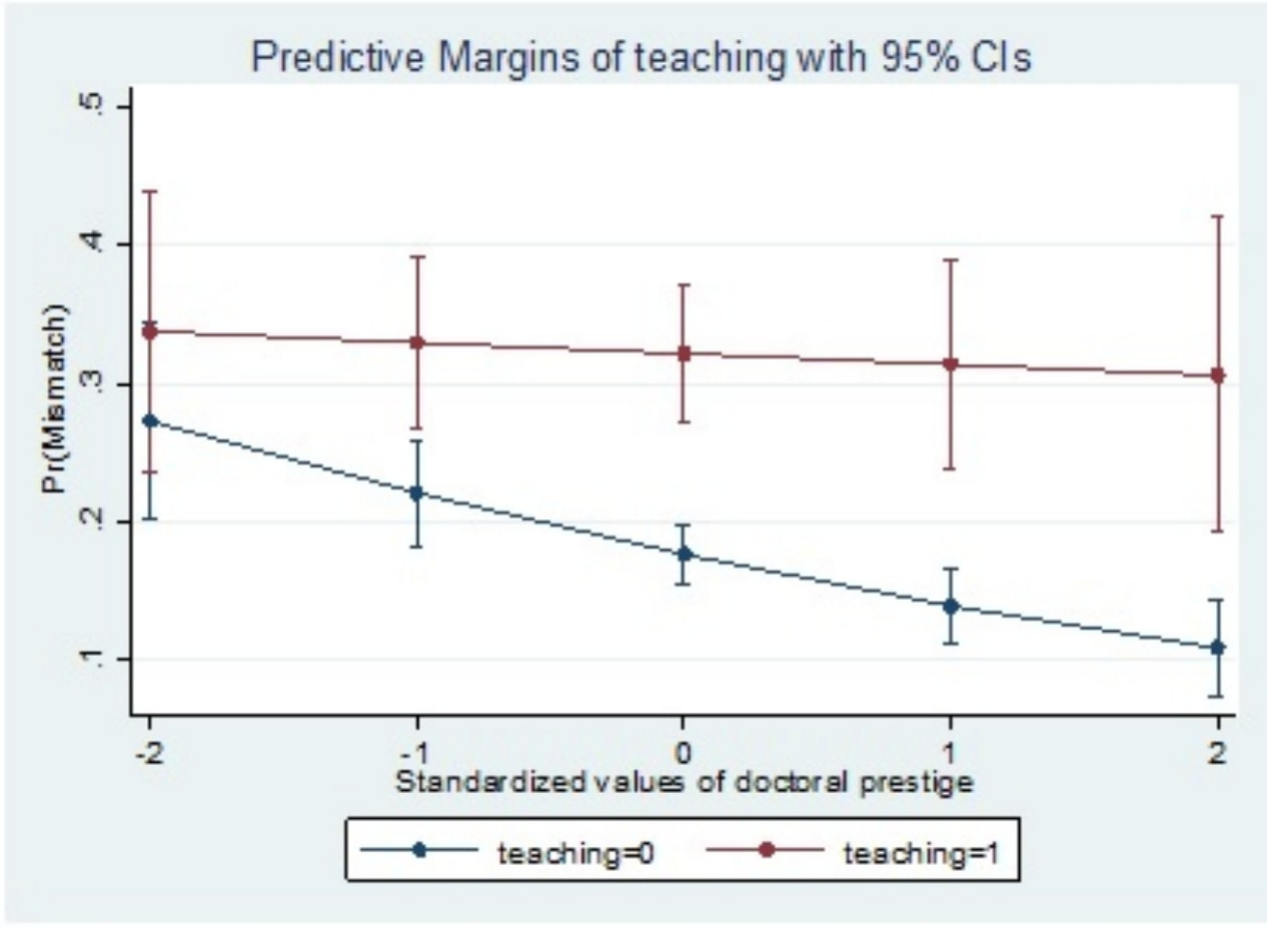
Career placement mismatch and institutional prestige.

Overall, prestige matters. At low levels of doctoral prestige (Fig 4), at least some individuals are more likely to be mismatched in their academic career placement regardless of their preference. For faculty with doctorates from more prestigious institutions, individuals with research preferences are unlikely to be mismatched, but individuals with teaching preferences remain statistically more likely to be mismatched. This seems to be consistent with previous findings [6] that professors from prestigious backgrounds who have a teaching preference often experienced higher levels of dissatisfaction in research oriented jobs.

### The role of prestige and advisors in career placement

Turning to our primary explanatory models, we examine the likelihood of placement in either a research or teaching oriented institution, and then specifically at placement in specific Carnegie Classified institutional types. We first conduct our analysis on our full sample, and then partition the data to run the models separately for men and women. We present marginal effects for ease of interpretation here: *each presented coefficient represents the percent change in likelihood of a particular outcome given a unit change in the independent variable*. Since these are marginal effects, the results of interaction terms are already shown in terms of their joint impact and significance (see supplemental materials for tables with relative risk ratios and separate terms for each of the variables that affect the interaction, plus interaction terms). For the interaction between doctoral prestige and preferences and the interaction between advisor sponsorship and preferences, the marginal effects are presented for respondents based on whether their initial academic career placement was research or teaching focused.

Table 2 presents the average marginal effects of the multinomial logit model applied to the full sample of faculty in the four disciplines included in our study. Our results show that teaching preference affects career placement in some cases. Consistent with the job search strategies noted earlier, it is not surprising that individuals with a teaching preference are 45% less likely to hold a position in a Research Extensive institution (*b* = -0.0456). Within teaching institutions, those with a teaching preference are almost twice as likely to be placed in a Master’s institution rather than a liberal arts college (30% versus 15%) (*b* = 0.312 and *b* = 0.157 respectively). Note that the liberal arts colleges included in our sample are among the most competitive in the nation (Oberlin 50).

**Table 2:** Academic career placement: Average marginal effects, full sample.

	Respondent Current Institution											
	Research Oriented Institution						Teaching Oriented Institution					
	Research Extensive			Research Intensive			Master’s I &II			Liberal Arts		
	B	Sig.	SE	B	Sig.	SE	B	Sig.	SE	B	Sig.	SE
***Career Preference/Support***												
***Teaching Preference***	-0.456	***	0.034	-0.013		0.020	0.312	***	0.025	0.157	***	0.013
Doctoral Prestige* Research Preference	0.071	***	0.014	-0.036	***	0.012	-0.045	***	0.014	0.010		0.007
Doctoral Prestige* Teaching Preference	0.008		0.039	-0.011		0.020	-0.024		0.019	0.027	***	0.008
Advisor Sponsorship* Research Preference	0.038	**	0.018	-0.020		0.014	-0.014		0.010	-0.004		0.004
Advisor Sponsorship* Teaching Preference	-0.005		0.023	0.003		0.014	-0.003		0.020	0.005		0.011
***Doctoral Training Background***												
***Dissertation Award***	0.164		0.047	-0.088	*	0.044	-0.055		0.034	-0.022		0.019
***Year of PhD***	-0.005	***	0.001	0.001		0.001	0.005	***	0.001	0.000		0.000
***Discipline***												
***Biochemistry***	0.043		0.032	-0.057	*	0.025	-0.090	***	0.025	0.104	***	0.010
***Civil Engineering***	0.109	**	0.034	-0.021		0.023	-0.036		0.025	-0.053	***	0.016
***Mathematics***	-0.074	*	0.036	0.008		0.024	0.036		0.023	0.030	**	0.011
***Demographics***												
***Dependent Child at PhD Completion***	-0.020		0.033	0.011		0.022	0.009		0.022	0.001		0.010
***Female***	0.026		0.027	0.003		0.018	-0.060	**	0.019	0.031	***	0.009
***First Generation College Graduate***	0.048		0.030	0.005		0.020	0.008		0.020	-0.060	***	0.010
***African American***	-0.060		0.061	-0.027		0.041	0.067		0.036	0.020		0.021
***Hispanic***	0.055		0.056	-0.118	*	0.057	0.039		0.038	0.024		0.022
***Native American/Alaskan***	-0.132		0.126	0.128		0.103	0.034		0.088	-0.029		0.049
***Asian***	-0.035		0.030	0.002		0.022	0.0476	*	0.021	-0.015		0.013
***Other Race/Ethnicity***	0.258		0.296	-0.161		0.150	-0.100		0.198	0.004		0.037
***N***	2555			2555			2555			2555		

* p<0.05

** p<0.01

*** p<0.001

Results also show that the demographic patterns in career placement are generally reflective of national statistics, and are consistent with findings from prior studies. Female faculty in our sample are about 3% (*b* = 0.031) more likely to be employed at Liberal Arts institutions than any other institutional types [35], and 6% (*b* = -0.060) less likely to be employed at Master’s Institutions. Notably, there are no significant statistical gender differences with regards to likelihood of employment at research institutions. Regarding race and ethnicity, there are no significant differences regarding the likelihood of employment at different institutional types, with the exception of Hispanics being slightly less likely to be at Research Intensive institutions. Having children of dependent age at time of PhD is similarly associated with no significant differences in employment patterns.

Year of PhD, on the other hand, has a significant impact on the type of institution in which tenure track faculty are employed. An increase of one year in terms of PhD graduation date leads to an increase of 0.5% in terms of probability of working at a Master’s institution, and a 0.5% decrease in the probability of working at a Research Extensive institution. This indicates that younger faculty are substantially more likely to work at Master’s rather than Research Extensive institutions. With regards to family background, being a first generation college graduate significantly impacts where an individual will work. First generation college graduates are less likely to work at Liberal Arts colleges than at the other institutional types noted, regardless of preference.

In the job search process, individual accomplishments show visible importance. Results highlight the importance of the visibility of research done as a graduate student with regard to institutional placement: having won a best dissertation award increases the probability of working at a Research Extensive institution by nearly 16%. This underscores other findings about how an early and successful start in research activity is important to career research productivity [22].

Regarding our key variables of interest, results show that institutional prestige and advisor sponsorship reveal some interesting relationships. To understand how preference interacts with the range of doctoral advisor involvement in the job search process, as well as with institutional prestige, these relationships were captured through four interactive variables. When preference (research vs teaching) is interacted with institutional prestige and advisor sponsorship, results are significant. Each additional standard deviation in terms of advisor support increases the probability of working at a Research Extensive institution by almost 4% for those with a research preference (significant at the 0.05 level). Among those with a research preference, individuals who received a maximum score for advisor support are nearly 14% more likely to work at Research Extensive institutions than those who received the average advisor support. In other words, having an advisor who more broadly “sponsors” an individual makes it more likely that that individual will work at a Research Extensive institution if he/she preferred a research-focused institution.

Importantly, when considering the interaction of preference and advisor support, the marginal effects are not significant for those with teaching preferences. This indicates that advisor involvement is particularly important for advisee placement at Research Extensive institutions, but individuals who prefer a teaching oriented institution see no change in the odds of landing at their preferred institutional type based on advisor sponsorship. This result, while somewhat surprising, has a possible explanation. Since advisors are, almost by definition, faculty at research oriented institutions, they may simply lack the connections or resources to help their students with teaching preferences land at teaching institutions. This lack of connections at teaching oriented institutions was conveyed to the authors of this project in a number of informal interviews with faculty at teaching oriented institutions in preparation for this project, and is supported by our research findings.

If advisor sponsorship is only crucial for those who seek employment at Research Extensive institutions, doctoral institutional prestige appears to behave much more as a form of capital. The results suggest that individuals from prestigious universities seem to be able to “use” that prestige to gain positions at the most prestigious of the research-oriented institutions, as illustrated by the significant interaction between research preference and institutional prestige. For those with a preference for research oriented institutions, an increase of one standard deviation in institutional prestige increases the probability of gaining a position at a Research Extensive institution by a little over 7%, while decreasing the probability of working at a Research Intensive or Master’s institution by approximately 3 and 4%, respectively. Given the high variation in our institutional prestige measure, this means that someone from a top ranked institution is about 20% more likely to land at a Research Extensive institution when compared to the average. Meanwhile, for those with a teaching preference, an increase in one standard deviation in PhD institutional prestige leads to an increase of about 3% in the probability of landing at a Liberal Arts college.

### Gender and academic career placement

These results point to certain factors that affect an individual faculty member’s ability to gain a position consistent with their career preferences. A point for further consideration is the extent to which this varies across individuals. Given gender disparities in academic science [29,53,54], and to address potential endogeneity of preferences and gender effects, we partitioned the data and ran these models separately for male and female faculty. Results for each subsample are provided in Tables 3 and 4.

**Table 3:** Academic career placement: Average marginal effects, male faculty.

	Respondent’s Current Institution											
	Research Oriented Institution						Teaching Oriented Institution					
	Research Extensive			Research Intensive			Master’s I &II			Liberal Arts		
	*B*	*Sig*	*SE*	*B*	*Sig*	*SE*	*B*	*Sig*	*SE*	*B*	*Sig*	*SE*
***Career Preference/Support***												
***Teaching Preference***	-0.446	***	0.047	-0.025		0.027	0.327	***	0.034	0.144	***	0.016
Doctoral Prestige* Research Preference	0.069	***	0.018	-0.036	**	0.014	-0.038	**	0.017	0.004		0.007
Doctoral Prestige* Teaching Preference	0.023		0.056	-0.016		0.030	-0.023		0.027	0.015	*	0.009
Advisor Sponsorship* Research Preference	0.039	*	0.022	-0.024		0.018	-0.009		0.012	-0.007		0.005
Advisor Sponsorship* Teaching Preference	0.013		0.035	0.015		0.020	-0.026		0.029	-0.002		0.016
***Doctoral Training Background***												
***Dissertation Award***	0.177	**	0.058	-0.087		0.051	-0.079	*	0.039	-0.011		0.020
***Year of PhD***	-0.007	***	0.001	0.001		0.001	0.006	***	0.001	0.000		0.000
***Discipline***												
***Biochemistry***	0.076		0.043	-0.065	*	0.032	-0.101	**	0.032	0.091	***	0.012
***Civil engineering***	0.116	**	0.044	-0.032		0.029	-0.042		0.031	-0.042	*	0.018
***Mathematics***	-0.053		0.047	0.000		0.032	0.016		0.030	0.036	**	0.013
***Demographics***												
***Dependent Child at PhD***	-0.046		0.041	0.011		0.028	0.030		0.026	0.005		0.011
***First Generation College Graduate***	0.040		0.036	0.004		0.025	0.006		0.024	-0.050	***	0.011
***African American***	-0.019		0.073	-0.043		0.052	0.026		0.046	0.036		0.021
***Hispanic***	0.036		0.078	-0.076		0.073	-0.003		0.051	0.042		0.024
***Native American/Alaskan***	0.150		0.154	0.283	*	0.117	0.349	***	0.100	-0.782	***	0.059
***Asian***	-0.016		0.039	0.000		0.028	0.038		0.025	-0.023		0.016
***Other Race/Ethnicity***	0.649	**	0.241	-0.261		0.176	-0.414	**	0.158	0.025		0.044
***N***	1418			1418			1418			1418		

* p<0.05

** p<0.01

*** p<0.001

**Table 4:** Academic career placement: Average marginal effects, female faculty.

	**Respondent’s Current Institution**											
	Research Oriented Institution						Teaching Oriented Institution					
	Research Extensive			Research Intensive			Master’s I &II			Liberal Arts		
	*B*	*Sig*	*SE*	*B*	*Sig*	*SE*	*B*	*Sig*	*SE*	*B*	*Sig*	*SE*
***Career Preference/Support***												
***Teaching Preference***	-0.485	***	0.036	0.009		0.024	0.281	***	0.026	0.194	***	0.021
Doctoral Prestige* Research Preference	0.061	***	0.018	-0.029	*	0.018	-0.061	***	0.022	0.029	**	0.015
Doctoral Prestige* Teaching Preference	-0.010		0.037	-0.004		0.022	-0.033		0.020	0.047	***	0.012
Advisor Sponsorship* Research Preference	0.026		0.023	-0.011		0.018	-0.020		0.015	0.004		0.008
Advisor Sponsorship* Teaching Preference	-0.054	***	0.020	-0.015		0.017	0.051	*	0.020	0.018		0.018
***Doctoral Training Background***												
***Dissertation Award***	0.150	*	0.062	-0.120		0.066	0.031		0.054	-0.061		0.047
***Year of PhD***	-0.001		0.002	0.001		0.001	0.002		0.001	-0.002	*	0.001
***Discipline***												
***Biochemistry***	-0.023		0.041	-0.042		0.037	-0.078	*	0.038	0.143	***	0.021
***Civil engineering***	0.121	*	0.049	0.013		0.034	-0.069		0.044	-0.065		0.034
***Mathematics***	-0.111	**	0.038	0.026		0.027	0.061	*	0.027	0.024		0.020
***Demographics***												
***Dependent child at PhD***	-0.023		0.041	-0.042		0.037	-0.078	*	0.038	0.143	***	0.021
***First Generation*** ***College Graduate***	0.121	*	0.049	0.013		0.034	-0.069		0.044	-0.065		0.034
***African American***	-0.111	**	0.038	0.026		0.027	0.061	*	0.027	0.024		0.020
***Hispanic***	-0.023		0.041	-0.042		0.037	-0.078	*	0.038	0.143	***	0.021
***Native American/Alaskan***	0.121	*	0.049	0.013		0.034	-0.069		0.044	-0.065		0.034
***Asian***	-0.111	**	0.038	0.026		0.027	0.061	*	0.027	0.024		0.020
***Other Race/Ethnicity***	-0.023		0.041	-0.042		0.037	-0.078	*	0.038	0.143	***	0.021
***N***	1137			1137			1137			1137		

* p<0.05

** p<0.01

*** p<0.001

When the models are run separately for male and female faculty, results reveal that advisor sponsorship seems to function differently by gender. For faculty who prefer a research intensive environment, advisor sponsorship has a statistically significant and positive (4%) effect in placement in a Research Extensive institution for men, but *has no effect for women*. For faculty with a teaching preference, however, results are considerably different, particularly by gender. For female faculty who reported a teaching preference, advisor sponsorship appropriately reduces the probability of landing at a Research Extensive university by about 5% for each additional standard deviation, and increases the probability of landing at a Master’s institution (lower prestige teaching institution) by slightly more than 5%. Yet, advisor sponsorship has no effect for male faculty with a teaching preference. However, it has no effect on placement in the highly prestigious (teaching oriented) liberal arts colleges for either men or women. These results reveal a gendered component to advisor sponsorship, where it has a positive effect for men with research preferences, but a somewhat mixed effect for women with teaching preferences.

There is also a difference between male and female faculty in how the prestige of their doctoral institution aligns with preferences to impact placement. While doctoral prestige functions similarly for both men and women with a research preference in increasing the likelihood of their placement in a Research Extensive institution (and decreasing the likelihood of their placement in a Master’s institution), for women it has a bigger impact in terms of affecting the probability of landing a job at a liberal arts college, regardless of preference. Results show that even those female faculty who reported a preference for research oriented institutions are more likely to be employed at a liberal arts college if they come from a prestigious doctoral program. Not surprisingly, the effect is slightly stronger for those with a teaching preference.

Notably, and consistent with the full model, results show no gender effects in how individual preferences for a teaching intensive environment affect institutional type placement. Individual accomplishments, in the form of a dissertation award, also function similarly for men and women, in impacting the likelihood of placement in a Research Extensive institution. Taken together, these results collectively indicate a world where doctoral prestige and advisor involvement work through mechanisms that are gendered, supporting placement for female faculty in teaching oriented institutions (but not high prestige liberal arts institutions) and male faculty in research oriented ones.

#### Advisor involvement and endogeneity

One possibility that might explain our results with regard to the impact of advisor involvement is the possibility of endogeneity in terms of advisor support. t is, advisor support may be conditioned on career preferences. While we cannot definitely rule that possibility out, evidence suggests that this is not the case. A t-test reveals no statistically significant difference in terms of advisor sponsorship across preferences. Likewise, if we estimate a regression analysis using all the variables from the full model with advisor sponsorship as a dependent variable, the results show an insignificant coefficient for teaching preference. There are, however, gender differences, and women report receiving less advisor sponsorship than men, which is consistent with other research that has found advisors being less willing to collaborate with female advisees, for example [22].

While there may not have been differences in terms of advisor sponsorship across preferences, we do have evidence of differences in terms of advice provided to respondents, as shown in Table 5.

**Table 5:** Respondent reported advisor experiences.

	**% respondents who reported initial academic career preferences as. . .**	***Advisor suggested a…***			
		research intensive position	teaching intensive position	less competitive position	more competitive position
*Male Faculty*	Research	44.47%	9.89%	4.13%	6.70%
	Teaching	28.71%	28.57%	2.57%	12.00%
*Female Faculty*	Research	43.52%	6.42%	5.70%	8.36%
	Teaching	28.44%	28.00%	2.67%	17.33%

Advisors are more likely to suggest research intensive positions, and rarely advise seeking teaching intensive positions, to those with a stated preference for research oriented jobs. For those interested in teaching positions, however, the advice is evenly split. That is, for those with a stated teaching preference, the advisor is just as likely to recommend a teaching oriented position as they are to recommend a research oriented position. The result here is that while there may not be significant differences in terms of reported things (letters, calls, etc.) provided by an advisor during their job search, there are differences in terms of what types of positions advisors recommend. Interestingly enough, and unlike our full models, there are very few differences in terms of gender. The only one above to be significant in a t-test is related to advisors suggesting more competitive positions than the respondent was interested in. Women with a teaching preference are more likely to report that their advisor advised them to seek a more competitive position than men with a teaching preference.

As a result, gender differences in terms of actual employment outcomes and preferences seem to be based not on reported levels of advisor sponsorship or advisor reactions to those preferences, but on factors beyond the scope of our study. For example, there may be qualitative differences in terms of advisor sponsorship (e.g., advisors may be providing less glowing letters of recommendation for women seeking research positions than for men with similar preferences) or to external perception of that sponsorship (e.g., search committees may be less trusting of letters of recommendation for women seeking research positions or men seeking teaching positions). We cannot discern between these possible causes, though recent research [55] provides some evidence for the latter hypothesis.

## Discussion

The main contribution of our work is that it highlights factors that may result in a career mismatch in the academic marketplace, some of which vary by gender. As we have noted, not all PhD scientists are interested in intensive academic *research* careers. A range of personal and professional factors shape academic career preferences. Our results provide meaningful progress towards addressing our research questions: What factors determine an individual’s ability to realize their academic career goals, particularly in the ability to select a more research or teaching centric academic position? Does preference alone determine career placement?

Understanding the factors that matter in achieving one’s preferred career placement is relevant to investigating inequities in the workforce and placement process, but also extends to other career-related issues. The general occurrence of a mismatch between one’s job and a variety of individual characteristics has been explored extensively. Such mismatches can occur between a job and an employee’s education level, educational background, preferred work schedule, skills, and/or interests. The fit of an individual to their position relates to several positive work-related outcomes, including self-efficacy, job satisfaction, and attraction to the organization and intentions to accept a job offer within the context of the job application process [56–58]. Specifically in the academic workplace, a host of negative consequences has been associated with such mismatches, including reduced income, increased turnover, and reduced job satisfaction [59,60].

The meaningful role that doctoral advisors play in the academic job search of a newly minted PhD is expected. Our results show that doctoral institutional prestige and advisor involvement in the job search process are substantially important in academic careers, but they are important in very different ways. Advisors play a key role for those wanting to pursue careers at research oriented universities. This seems to be in part because of a strong relationship between advisor involvement and preference (i.e., there seems to be a degree of endogeneity between the type of support received from advisors and preference). But even taking this into account, advisor support is still important when we look only at those with a research preference. This provides evidence that while advisors play an important part as “sponsors” within academia, their contribution is restricted to the institutional type that they themselves are familiar with. This is also consistent with studies of PhD recipients with interest in non-academic careers, which also note a lack of advisor support [1,25]. Advisor influence seems to disappear when we look at those individuals who would prefer a teaching oriented career, even for those who graduate from the most prestigious teaching institutions.

If advisor influence is limited to those who want to pursue a research oriented career, doctoral prestige behaves much more like a form of capital [41]. When Bourdieu discussed the forms of capital, he used the capital analogy as something that could be deployed to achieve a certain social status. Within academia, prestige seems to work that way: its effect is contingent on how the individual “employs” it. Those who prefer a teaching oriented career are able to transform that prestige into a greater likelihood of teaching at Liberal Arts colleges, where our data have shown that individuals are more likely to report satisfaction with their teaching obligations (see Materials and Methods section). And for those with research aspirations, prestige opens doors at the more resource rich Research Extensive institutions. Besides differentiating between the impact of advisors and institutions, this finding is remarkable because it indicates that previous research has actually underestimated the importance of prestige in the academic labor market. As noted previously, most research on academic prestige has found that within Research Extensive institutions there is a caste [12] that tends to come from the same elite institutions. We have shown that those from highly prestigious institutions who have a teaching preference are more likely to work at Liberal Arts colleges. Alumni from elite departments that land at places other than top ranked research institutions may do so by following their preferences. If not for those preferences, we could imagine a much more significant dominance of these elite departments within research oriented universities and colleges.

A few caveats are in order. As is the case in most existing research on academic careers, our study suffers from survival bias. While our sample goes beyond many previous studies by including different institutional types, it is still limited by the fact that we only have data on tenured and tenure track faculty. Research on individuals who move into non-tenure track academic positions (e.g. research scientists), whether by choice or necessity, is sorely needed in order to understand the full breadth of how preferences and various support mechanisms play a role in the academic marketplace. It may very well be the case that the impacts of prestige and advisor sponsorship are even greater than estimated here if we consider those who involuntarily leave academia. Our results are hampered by our lack of data on those who exit academia all together, or do not choose it in the first place. Given this, our results are specific to those who persist in the academic workforce.

Related, we also lack data on the institutional culture and support mechanisms for career choices in our respondent doctoral institutions. While advisor support may be important, other factors such as support for non-research careers from other faculty or career/teaching related resources on campus may impact career preferences and direction. Given this, we are not able to examine the cultural and other factors that matter in early career placement.

Another important caveat is that we only have data from one point in time, and the information we have on preferences is based on individual recollection. To the extent that it is possible, we have tried to address that by confirming our results with different samples. While we presented results from a sample with a full range of career stages, our results are substantively consistent if we focus only on junior faculty or on those who are on their first tenure track appointment. Still, there is always the possibility that people may remember their initial career preferences inaccurately. Thus, some caution should be observed in our interpretation of results. More comprehensive qualitative studies might be able to shed some light on these issues.

## Materials and methods

### Statistical methods

To contextualize our research question and subsequent analysis, we first provide a descriptive analysis of the initial career preferences and current placement of our survey respondents. Using frequencies and a comparison of means we examine the extent to which a mismatch of preferences and placement exists. We also use a descriptive model which allows us to control for various demographic and other background factors in explaining mismatch.

Next, to test the hypotheses noted above, we use a multinomial logit model[61] to address how career preferences, institutional prestige and advisor support explain career placement. Multinomial logit models are among the most popular methods for analyzing issues where discrete choices are at play. By using this method, we can estimate how different variables affect the probability of a given outcome for each observation. This approach is ideal for our purposes, given that our outcomes exist in the form of a nominal variable with four mutually exclusive possibilities (i.e., employment at a Research Extensive, Research Intensive, Liberal Arts or Master’s institution). Our models are weighted by sampling probability, as discussed below. To deal with the issue of research versus teaching career placement preference, we ran three different models. One model included the full sample, and had teaching preference as an independent variable, which was also interacted with the key independent variables. The other two were restricted by gender, to understand the ways in which different factors affect men and women. We are interested in gender differences given overall disparities in STEM careers, including that women faculty are employed in Research Intensive Institutions at a slightly lower rate than are men [35].

### Data and variables

Our data come from a large National Science Foundation-funded project (NETWISE II). The primary data collection for this project involved the implementation of an extensive survey of STEM faculty in the United States. A significant concern of the project was to address academic career distinctions by gender and race/ethnicity. Given this, four STEM fields were selected for inclusion: biology, biochemistry (high female representation), civil engineering (transitioning female representation), and mathematics (lower levels of female representation). Another purpose of the project was to understand career variations across the broader academic STEM workforce. Therefore, the population included all tenured/tenure-track faculty in our selected disciplines from not only the research-centric institutions (Carnegie Classified Research Extensive and Research Intensive institutions), but also teaching-centric (Historically Black Colleges and Universities (HBCUs), a cluster sample of Master’s I/II and Hispanic Serving Institutions (HSIs), Women’s Colleges, and the Oberlin 50 baccalaureate) institutions offering degrees in the four target disciplines. The institutional types included here account for nearly 28% of all institutions of higher learning in the United States, and nearly 75% of all 4 year institutions. The survey and protocol were approved by the Institutional Review Board as part of Human Subjects protection. Once the clustered institutional samples were selected, we conducted a sampling procedure in order to stratify across institutional type, faculty rank and discipline, and oversample for gender, resulting in a final sample of 9,925 (38% of the original sampling frame).

The survey addressed a broad set of items relevant to the study of academic careers in STEM. Sections included individual background, job search experiences, early career preferences, relationship with advisor and mentors, positions held and other advancements, research, teaching and professional activities, and other professional experiences. The survey was implemented online and had a total unweighted response rate of 42%, with 4,195 completed or partially completed surveys submitted. Because we are interested in career placement issues and career preferences, we are focusing on individuals who reported having an initial preference for academic careers (as opposed to industry or government) with either a research oriented or teaching oriented focus, for a total of 2,670 respondents used in our analysis. To account for the weighting procedure described above with regards to the sample, all our models use appropriate sample weights. Table 6 presents the number of institutions with at least one respondent included in our sample, by institutional type.

**Table 6:** University and college institutional types: Population, sample, and represented.

	**Number of Institutions in the U.S, by Carnegie Classification**	**Percent of Institutions in the U.S, by Carnegie Classification**	**Number of Institutions in Survey Sample**	**Number of Institutions Included in Models**
***Doctoral/Research—Extensive***	151	3.8%	144	135
***Doctoral/Research—Intensive***	110	2.8%	94	94
***Master's I & II***	611	15.5%	170	160
***Baccalaureate—Liberal Arts***	228	5.8%	69	67
***Baccalaureate—General***	321	8.1%	2	—
***Baccalaureate/Associate's***	57	1.4%	—	—
***Associate's***	1,669	42.3%	—	—
***Specialized Institutions***	766	19.4%	8	—
***Tribal Colleges and Universities***	28	0.7%	—	—
***Total***	**3,941**	**100%**	**487**	**456**

NOTE: Frequencies do not reflect changes made after 01/30/2001.

Source: Carnegie, 2004

### Dependent variable

#### Institutional type

The dependent variable in our models is the institutional type at which the respondent is currently employed. Using the 2000 Carnegie Classification system, our sample includes Carnegie Classified Research Extensive, Research Intensive, Master’s I & II and Liberal Arts colleges [19]. We code Research Extensive and Research Intensive to be “research oriented” institutions, while Master’s and Liberal Arts institutions are coded as “teaching oriented”. (Note that the Liberal Arts schools include not only the Oberlin 50 but also HBCU and Women’s Colleges from our sample). The rationale here is that the target disciplines in the Research Extensive and Research Intensive institutions are typically doctoral degree granting and research focused, whereas the Master’s I & II and Liberal Arts institutions are more teaching focused. To illustrate these assumptions, Table 7 includes basic descriptive statistics from the survey data regarding teaching and research involvement at each institutional type. Respondents in Research Extensive institutions report spending more time on research and less time on teaching as compared to their peers in Research Intensive institutions; furthermore, faculty from both of these research-centric institutions provide reports that are notably different from those of the Master’s I & II and Liberal Arts faculty. These descriptive data demonstrate varied expectation and also satisfaction with research and teaching loads across this set of schools.

**Table 7:** Respondent research and teaching expectations, by institutional type:.

	Research Extensive 143 Institutions (n = 1068)	Research Intensive 94 Institutions (n = 759)	Master’s I & II 165 Institutions (n = 1152)	Liberal Arts 69 Institutions (n = 637)
*Recoded as*:	Research Focused		Teaching-Focused	
***Share of sample***	29.54%	20.99%	31.86%	17.62%
***% preferred teaching oriented institution***	5.73%	16.10%	46.19%	31.92%
***% preferred research oriented institution***	45.28%	24.45%	21.61%	8.66%
***Average % of time spent on teaching***	30.74%	42.00%	54.45%	60.78%
***Average % of time spent on research***	49.70%	36.96%	23.18%	20.03%
***% who are “very satisfied” with teaching opportunities***	15.28%	21.16%	27.17%	43.16%
***% who report being “required to seek external research funding for promotion”***	91.42%	76.72%	55.04%	44.19%

### Independent variables

#### Doctoral institutional prestige

Existing research has demonstrated that the key determinant of prestige rankings within academia is the centrality of the institution within hiring networks (e.g.,[12,40]). High prestige institutions are institutions that hire and place their graduate students at other similarly central institutions. We measure doctoral prestige by estimating a department's eigenvector centrality in hiring networks. To do this, we created a network that linked PhD institutions with hiring institutions, and estimated eigenvector centrality using social network analysis software. PhD institution was reported by respondents in the survey, and current institution was identified in our population development via internet search, and verified by survey respondents. Centrality is measured as the reciprocal of the average shortest distance between one institutional node and all others, where the smaller the average shortest distance, the higher the eigenvector centrality. We use eigenvector centrality because this measure is highly correlated with existing survey-based prestige measures [40]. We use undirected networks to take into account that a substantial number of institutions involved in our sample do not have graduate programs, as it allows us to assign a greater degree of centrality if they frequently hire from other high prestige places.

We use this approach in order to solve the challenges presented by our institutionally and disciplinarily diverse data set. Survey based measures of departmental prestige (i.e., measures where prestige values are determined through a survey of academics in the field), such as the 1995 NRC rankings, the current US News and World report rankings or the QS World University Rankings will frequently not cover all institutions, nor all fields. Using these measures would not allow us to compare across the various disciplines in our study, as well as many of our sampled institutions, thereby severely impacting our usable sample.

Our choice of institutional centrality as a measure of prestige does not appear to produce any different results than alternative measures would provide. Our measure of centrality is highly correlated with existing field and institutional-type specific rankings, such as the National Research Council’s (NRC) 1993 survey of doctoral program prestige, published in 1995 [62]. To check for any potential bias from our selected approach, we analyzed the NRC and other rankings against our eigenvector centrality measure, and found them to be highly correlated (Table 8). Further, all our results are consistent in terms of size and significance of effects, regardless of whether we use eigenvector centrality measures or NRC measures.

**Table 8:** Prestige correlations for survey sample.

	***Survey Overall Centrality***	***US News***	***Survey Discipline Centrality***	***NRC 1995 Rankings***	***QS Academic Reputation***
***Survey Overall Centrality***	1.000				
***US News***	0.735	1.000			
***Survey Discipline Centrality***	0.801	0.651	1.000		
***NRC 1995 Rankings***	0.652	0.902	0.610	1.000	
***QS Academic Reputation***	0.665	0.810	0.502	0.771	1.000

As the correlation results show, our measure of institutional centrality correlates with existing prestige measures between 0.65 and 0.735. Detailed results on this analysis may be found in the S1–S3 Appendices. These include the average marginal effects for the full models using each of the different prestige measures mentioned above (1995 NRC rankings, current US News and World Report rankings and QS World University Rankings).

As an example, our measure finds that the most central (and as such most prestigious) places in our sample are UC-Berkeley for research institutions, California Polytechnic State University-San Luis Obispo for Master’s institutions, and Bucknell, Denison and Swarthmore (tied) for baccalaureate colleges, which are fairly consistent with published rankings.

#### Teaching (career placement) preference

To account for individual career placement preferences, we include a teaching preference dummy variable. This variable is based on the survey question: “As you were finishing your PhD, what was your preferred career choice?” The choices were mutually exclusive, and included “tenure track position at a research intensive institution” and “tenure track position at a teaching intensive institution,” "position in industry," "position in government," or "non-tenure track academic position." The teaching preference dummy is based on the latter. Given that the question asks specifically about preferences when the respondent first went on the market, it is entirely possible that some respondents have changed their preferences since that time, or that there is some other form of recall bias, introducing some bias or noise in our models given that the dependent variable is about current employment. Nonetheless, we are confident that our results are robust: our results remain consistent even if we reduce our sample to only pre-tenure faculty, or to only faculty who are still in their first tenure track appointment.

#### Advisor sponsorship

Regarding advisor sponsorship, we focus on active and tangible ways that advisors supported respondents in their job search. While co-authorship with early career researchers has been demonstrated to be important in later career productivity[22], other activities demonstrate active engagement in the job search process. We focus on the relational characteristics of the advisor-advisee relationship, which capture the extent to which an advisor is invested in a particular candidate. Our variable is based on respondents’ indication that their advisors did the following for them in their initial job search:

- Wrote recommendation letters
- Made phone calls on their behalf
- Defended career choice with others
- Gave advice on how to negotiate

We transformed these four variables into a single measure that captures the extent to which these resources were or were not provided. To do this, we conducted a principal component factor analysis of the 4 binary items, and created a single standardized measure of advisor sponsorship (Eigenvalue = 1.45). We used polychoric correlations (PCA)[63], as is appropriate for combining discrete and binary variables into a measure that captures a concept as a single measure. Unlike a simple summative variables, PCA allows variables to be weighted differently, acknowledging the variation in importance of these different factors. The principal component estimated here explains 0.53, or about 53%, of the variation in the 4 items. Details on the scoring of the principal factor are available in S1–S3 Appendices.

To guard against the possibility of recall bias regarding advisor involvement given the variation in time lapsed since our respondents would have had this interaction, we took two different strategies. First, we compared our measure of advisor involvement to a question regarding co-authorship with one's advisor (less likely to be forgotten as an event). Correlations between our measure of advisor involvement and a variable regarding advisor co-authorship are not statistically significantly different for respondents with pre-1995 PhDs and post-1995 PhDs. Additionally, our results with regards to advisor involvement are substantially similar if we restrict our sample to assistant and associate professors, with the only difference being that for male faculty with a research preference the coefficient becomes insignificant, though still with the same sign. For female faculty, the results are substantially the same.

### Control variables

Our models include a number of additional control variables. We include basic demographic information such as race, ethnicity, gender and whether the respondent had a child of dependent age at the time of their PhD (though we have no information on custody). Additionally, we include discipline-specific dummy variables to control for any field variations. We also control for year of PhD, in order to control for seniority and stage of one’s career. To account for individual strengths which may matter in the job market, we also include a dummy variable that indicates whether the respondent has received a dissertation award. This item is included as a way of controlling for respondent research visibility as a graduate student, and separates ascriptive measures such as departmental prestige and advisor involvement from personal qualifications. Finally, we also introduce a dummy variable meant to capture cultural capital, or the “cultural competence” that individuals accumulate as a result of their affiliation or experience [41]. Cultural capital has been found to have some impact on advisor relationships; for example, Pinheiro, Melkers and Youtie [22], found that individuals with faculty parents are more likely to collaborate with their advisors on publications. Thus we included a measure of whether the respondent is a first generation college graduate. The variables included in our models are summarized in Table 9. One potentially important factor that we are not able to take into consideration is marital status at the time that the respondent was on the job market. While we do know marital status at the time of the survey, it is not possible to address this important possible constraint on job seeking behavior. While not shown here, earlier in our analysis we did include the binary variable “never married” (8% of our sample), which was not significant across all our models.

**Table 9:** Descriptive statistics for independent variables.

**Variable**	**Percentage**	**SD**	**Minimum**	**Maximum**
***Prestige***	0%	1	-1.48	2.63
***Advisor Sponsorship***	0%	1	-1.33	3.74
***Teaching Preference***	33%	0.47	0	1
***Dependent child at PhD***	26%	0.44	0	1
***First Generation College Graduate***	28%	0.45	0	1
***Female***	43%	0.50	0	1
***African American***	7%	0.26	0	1
***Hispanic***	6%	0.24	0	1
***Native American***	1%	0.08	0	1
***Asian***	23%	0.42	0	1
***Other Race/Ethnicity***	1%	0.10	0	1
***Average Year of PhD***	1993	10.73	1958	2011
***Dissertation Award***	6%	0.24	0	1
***Biology***	34%	0.47	0	1
***Biochemistry***	17%	0.38	0	1
***Civil Engineering***	19%	0.39	0	1
***Mathematics***	28%	0.45	0	1

## Supporting information

S1 Appendix Using alternative prestige measures. (PDF)

S2 Appendix Scoring advisor support. (PDF)

S3 Appendix Relative risk ratio tables. (PDF)
