# Supplementary material for: Take me where I want to go: Institutional prestige, advisor sponsorship, and academic career placement preferences
Source: PLoS One. 2017 May 11;12(5):e0176977. doi: 10.1371/journal.pone.0176977 (PMC5426638; doi:10.1371/journal.pone.0176977)
Supplement: S1 Appendix — (PDF) [file pone.0176977.s001.pdf]

Appendix S1: Using Alternative Prestige Measures

There are no perfect measures of academic prestige, and most existing measures have specific drawbacks. In this article, we measure doctoral prestige by estimating a department's eigenvector centrality in hiring networks. To do this, we created a network that linked PhD institutions with hiring institutions, and estimated eigenvector centrality using social network analysis software (UCINET). In particular, we measured centrality as the reciprocal of the average shortest distance between a node and all others. A very central department (one that places a lot of students at other very central departments) will have a shorter "path" to reach all other departments in the network. As such, the smaller the average shortest distance, the higher the eigenvector centrality. We use eigenvector centrality because recent research [1] has demonstrated that this measure is highly correlated with existing survey based prestige measures.

The reason we prefer to use this measure here, instead of any number of existing survey based measures of departmental prestige, is driven by the institutional and disciplinary diversity of our sample, and the significant loss of data if we were to use other measures. Survey based measures of departmental prestige (i.e., measures where prestige values are determined through a survey of academics in the field), such as the 1995 NRC rankings, the current US News and World report rankings or the QS World University Rankings will frequently not cover all institutions, or not cover all fields. As a result, had we used any of these measures, our sample size would be severely impacted, and resulting possible bias in our results. While our measure is survey based and thus subject to any potential biases that are present in our sample, it is important to highlight that our measure correlates highly with existing measures, and, more importantly, that all our results are consistent, regardless of which measure we use.

Also included is an alternative measure of centrality, one which generates discipline specific centrality. In order to confirm these correlations, we also created a correlation matrix (Table S1.1) that is based on our institutional sample (i.e., each observation is an institution).

Table S1.1: Prestige correlations for Institutional Sample:

	QS Academic Reputation	US News Civil Engineering	US News Math	US News Biology	NRC Biochemistry	NRC Biology	NRC Civil Engineering	NRC Match	Survey Overall	Biology Centrality	Biochemistry Centrality	Civil Engineering Centrality	Math Centrality
QS Academic Reputation	1.00												
US News Civil Engineering	0.71	1.00											
US News Math	0.88	0.80	1.00										
US News Biology	0.81	0.77	0.87	1.00									
NRC Biochemistry	0.64	0.61	0.75	0.85	1.00								
NRC Biology	0.62	0.53	0.73	0.84	0.88	1.00							
NRC Civil Engineering	0.81	0.90	0.83	0.75	0.70	0.59	1.00						
NRC Math	0.80	0.72	0.95	0.82	0.74	0.72	0.81	1.00					
Survey Overall Centrality	0.67	0.82	0.73	0.65	0.56	0.53	0.76	0.67	1.00				
Biology Centrality	0.46	0.59	0.56	0.59	0.47	0.48	0.48	0.49	0.82	1.00			
Biochemistry Centrality	0.60	0.64	0.63	0.55	0.44	0.42	0.65	0.55	0.80	0.60	1.00		
Civil Engineering Centrality	0.38	0.74	0.43	0.38	0.34	0.28	0.65	0.39	0.72	0.49	0.45	1.00	
Math Centrality	0.50	0.51	0.60	0.53	0.48	0.49	0.53	0.57	0.73	0.59	0.53	0.39	1.00

One interesting aspect to note is that overall centrality has higher correlations than discipline specific centrality even with discipline specific measures of prestige, pointing to possible halo effects.

All our models have been estimated using alternative prestige measures, and our results are very consistent, if not nearly identical in many cases. Further, all results are consistent with the results presented in Table 1 of the main paper. Table S1.2 contains the results of our logistic regression models regarding mismatch:

Table S1.2. **Logit Regression: results with alternative measures of prestige, odds ratios reported**

	Discipline Specific Centrality			US News			QS Rankings			1995 NRC		
	Odds ratio	Sig	SE	Odds ratio	Sig	SE	Odds ratio	Sig	SE	Odds ratio	Sig	SE
**Career Preference/Support**												
**Teaching preference**	2.302	***	0.334	2.553	***	0.407	2.924	***	0.503	2.384	***	0.377
**Advisor Sponsorship**	0.907		0.073	0.913		0.085	0.938		0.092	0.938		0.082
**Teaching preference*advisor sponsorship**	1.039		0.139	1.009		0.151	0.961		0.155	0.983		0.147
**Teaching preference*doctoral prestige**	1.456	**	0.218	1.72	***	0.253	1.193		0.185	1.592	***	0.232
**Doctoral Training Background**												
**Doctoral Institution Prestige**	0.712	***	0.066	0.608	***	0.055	0.795	**	0.074	0.683	***	0.059
**Dissertation Award**	0.425	***	0.126	0.441	**	0.142	0.407	***	0.142	0.429	***	0.141
**Year of PhD**	1.002		0.006	0.997		0.006	1		0.006	1.001		0.006
**Discipline**												
**Biochemistry**	0.59	***	0.099				0.576	***	0.113	0.624	***	0.112
**Civil Engineering**	1.002		0.184	0.853		0.168	0.976		0.209	1.02		0.209
**Mathematics**	0.968		0.154	1.03		0.174	1.036		0.202	1.021		0.182
**`x**												
**Dependent Child at Year of PhD**	1.047		0.154	0.859		0.148	0.874		0.160	0.952		0.159
**Female**	1.025		0.132	0.981		0.145	1.039		0.160	1.078		0.152
**First Generation College Graduate**	1.118		0.158	1.067		0.172	1.186		0.198	1.078		0.169
**African American**	1.225		0.314	1.477		0.403	0.857		0.301	1.007		0.293
**Hispanic**	0.849		0.241	0.881		0.27	0.807		0.271	0.917		0.285
**Native American**	0.808		0.588	0.897		0.627	0.128	*	0.142	0.266		0.225
**Asian**	1.003		0.145	0.864		0.14	0.824		0.148	0.855		0.134
**Other Race/Ethnicity**	4.687	**	2.987	5.254	**	3.717	5.535	**	3.741	5.195	***	3.472
**Constant**	0.007		0.085	119.1		1571	0.0893		1.211	0.0683		0.896
N	2,555			1,921			1,884			2,214		
Log Likelihood	-3066.7164			-2542.364			-2178.7627			-2587.153		
Pseudo R2	0.054			0.075			0.073			0.062		

*** p<0.01, ** p<0.05, * p<0.1

Below are average marginal effects for full models using each of the different prestige measures mentioned above (1995 NRC rankings, current US News and World Report rankings and QS World University Rankings)[2–4].

Table S1.3 Average marginal effects for prestige variable, alternative specifications

	Research Extensive	Research Intensive	Master's I & II	Liberal Arts
	US News and World Report			
Research Preference	0.121***	-0.0518***	-0.0820***	0.0132*
	-0.02	-0.01	-0.01	-0.01
Teaching Preference	0.0257	-0.00988	-0.0446***	0.0287***
	-0.03	-0.02	-0.02	-0.01
N	1986	1986	1986	1986
	QS World University Rankings			
Research Preference	0.0621***	-0.0336***	-0.0545***	0.0260***
	-0.02	-0.01	-0.01	-0.01
Teaching Preference	0.00562	-0.00424	-0.0279*	0.0265***
	-0.03	-0.02	-0.02	-0.01
N	1956	1956	1956	1956
	1995 NRC rankings			
Research Preference	0.0844***	-0.0348***	-0.0694***	0.0198***
	-0.0147	-0.0105	-0.0112	-0.00723
Teaching Preference	0.0296	-0.00388	-0.0430**	0.0173**
	-0.04	-0.02	-0.02	-0.01
N	2276	2276	2276	2276
	Discipline Specific Centrality			
Research Preference	0.075***	0.030**	0.051***	0.007
	0.015	0.0132	0.017	0.007
Teaching Preference	0.022	-0.015	-0.025	0.018**
	0.038	0.019	0.019	0.008
N	2555	2555	2555	2555

As Table S1.3 here shows, there are some minor differences in regards to level of significance for Master's institutions for those with teaching preferences, but overall the take away point is the same. Also important to note that using any of these alternative measures of prestige would reduce our sample by nearly 400 observations, at a minimum.

It is important to note that for the purposes of checking the consistency of our measures, we use the 1995 NRC Rankings instead of the most recent ones because the most recent ones notably do not include any survey questions regarding program prestige, instead relying on regression models based on quantitative data. As a result, they are less a measure of prestige and more a measure of program quality based on quantitative criteria. The end result is that the recent NRC rankings do not present a single, quantitative score of prestige like its 1995 counterpart did, but instead a range of rankings based on the confidence intervals of the underlying regressions, making it impossible to reduce to a single value. As Baldi [5] and others have discussed, academic prestige is very "sticky," and despite the time gap the 1995 NRC rankings and current US News rankings (which were measured between 2014 and 2016 depending on discipline) are correlated at over the 0.843 level.

Finally, table S1.4 contains the centrality scores, both overall and discipline specific, based on our sample. A few words of caution are necessary when looking at any measure of prestige or centrality. First, while there is a tendency for the public at large to focus on ordinal aspects of prestige measures, it is substantially more useful to focus on the quantitative measures themselves. That is, focusing on ordinal aspects of prestige rankings (e.g., whether an institution should be ranked 2nd or 5th) generally revolves around microscopic differences in whatever measure of prestige that is being used. These can never be resolved satisfactorily in a statistical way. Much more productive is to focus on the magnitude of these differences. For example, it may be impossible nor realistic to resolve who is number 1 or number 2 in the rankings, but most rankings will agree in terms of which institutions are, say, in the top 20 versus which are below rank 50. Second, our own measures may contain a result or two that may seem anomalous. It is not our intention to provide a definitive ranking mechanism. All prestige and centrality measures have their limitations. NRC data last collected information on prestige in 1995. US News and World Report does not report the results of programs with a prestige measure lower than 2, and does not include all disciplines (biochemistry, for example, is grouped with biophysics and structural biology as a subspecialty of biology, and is not ranked in the same way as the other disciplines in our sample). QS World University Rankings do not distinguish between graduate and undergraduate education, nor between disciplines within universities. Our measures here have the major benefit of maximizing our number of data points, but as with any survey data, is subject to a number of limitations. More important to note here, once more, is the fact that regardless of which measure is being used all our results are consistent through all the models.

Table S1.4: Eigenvector Centrality measures by Institution:

Institution Name	Overall Centrality	Biology Centrality	Biochemistry Centrality	Civil Engineering Centrality	Math Centrality
University of California - Berkeley	0.016	0.021	0.021	0.032	0.026
University of California - Davis	0.012	0.017	0.025	0.015	0.007
Cornell University	0.011	0.011	0.018	0.014	0.008
University of Wisconsin - Madison	0.011	0.009	0.018	0.002	0.022
University of Michigan - Ann Arbor	0.011	0.01	0.012	0.019	0.007
Stanford University	0.011	0.009	0.02	0.022	0.007
Massachusetts Institute of Technology	0.01	0.001	0.023	0.009	0.016
Yale University	0.01	0.012	0.019	0	0.016
University of Texas at Austin	0.009	0.008	0.008	0.025	0.005
University of California - San Diego	0.009	0.012	0.02	0.002	0.005
Purdue University	0.009	0.007	0.011	0.024	0.007
University of California - Los Angeles	0.009	0.012	0.01	0.009	0.007
University of Washington-Seattle Campus	0.009	0.014	0.007	0.012	0.008
Harvard University	0.008	0.014	0.01	0.002	0.008
Michigan State University	0.008	0.008	0.013	0.008	0.006
University of Illinois at Urbana-Champaign	0.008	0.006	0.009	0.015	0.007
Texas A & M University	0.008	0.006	0.003	0.023	0.003
Johns Hopkins University	0.007	0.006	0.011	0.01	0.003
Pennsylvania State University - Penn State Main Campus	0.007	0.004	0.013	0.015	0.008
University of Colorado at Boulder	0.007	0.006	0.017	0.01	0.001
SUNY at Stony Brook	0.007	0.01	0.002		0.011
Georgia Institute of Technology - Main Campus	0.007	0.004	0.006	0.022	0.002
University of Maryland - College Park	0.006	0.005	0.013	0.004	0.006
Princeton University	0.006	0.004	0.004	0.003	0.01
University of New Mexico-Main Campus	0.006	0.008	0.005	0.003	0.009
Duke University	0.006	0.015	0.005	0.004	0.002
University of Pennsylvania	0.006	0.005	0.008	0.002	0.005
University of Minnesota - Twin Cities	0.006	0.004	0.006	0.009	0.014
Northwestern University	0.006	0.001	0.008	0.017	0.005
The University of Texas at San Antonio	0.005	0.009		0.006	0.001
Florida International University	0.005	0.007	0.007		0.001
University of Connecticut	0.005	0.005	0.004	0.006	0.004
The University of Texas at El Paso	0.005	0.005		0.007	0.008
California Institute of Technology	0.005	0.002	0.014	0.008	0.002
University of Virginia	0.005	0.005	0.004	0.01	0.002

Institution Name	Overall Centrality	Biology Centrality	Biochemistry Centrality	Civil Engineering Centrality	Math Centrality
Iowa State University	0.005	0.004	0.003	0.012	0.005
University of Chicago	0.005	0.008	0.007		0.005
New Mexico State University	0.005	0.007	0.005	0.002	0.007
Ohio State University	0.005	0.003	0.004	0.006	0.01
University of California - Santa Barbara	0.005	0.004	0.006	0	0.007
University of California - Irvine	0.005	0.005	0.008	0.007	0.001
University of North Carolina at Chapel Hill	0.004	0.005	0.006	0.001	0.002
University of Iowa	0.004	0.002	0.002	0.009	0.005
University of Southern California	0.004	0.005	0	0.007	0.001
Howard University	0.004	0.003	0	0.005	0.007
University of Tennessee	0.004	0.004	0.005	0.005	0.001
Virginia Polytechnic Institute and State University	0.004	0.005	0.003	0.011	0.001
Arizona State University	0.004	0.004	0.002	0.007	0.001
Louisiana State University and Agricultural & Mechanical College	0.004	0.005		0.009	0.005
University of Massachusetts-Amherst	0.004	0.002	0.005	0.004	0.003
University of Nebraska - Lincoln	0.004	0.001	0.005	0.006	0.004
University of Delaware	0.004	0.001	0.004	0.008	0.004
Colorado State University	0.004	0.002	0.008	0.005	0.001
University of Alaska Fairbanks	0.004	0.006	0.007	0.003	0.001
Brown University	0.004	0.003	0.002	0.002	0.01
University of Notre Dame	0.004	0.003	0.009	0.006	0.002
Wesleyan University	0.004	0.005	0.006		0.003
North Dakota State University - Main Campus	0.004	0.003	0.006	0.007	0.003
Columbia University in the City of New York	0.004	0.002	0.007	0.001	0.005
University of Kansas	0.004	0.006	0.005	0.007	0.002
Indiana University Bloomington	0.004	0.005	0.004		0.004
Brigham Young University	0.003	0.002	0.007	0	0.003
University of California - Riverside	0.003	0.002	0.009		0.001
Union College	0.003	0.006	0.002		0.004
Central Michigan University	0.003	0.003		0.003	0.006
University of Georgia	0.003	0.008	0.001	0	0.002
West Virginia University	0.003	0.001		0.009	0.003
Missouri University of Science and Technology	0.003	0.001	0	0.013	
The University of Texas at Arlington	0.003	0.001		0.004	0.006
University of Maryland - University College	0.003	0.001	0.004	0.006	0.001
University of Arizona	0.003	0.003	0.007	0.002	0.001
The University of Montana	0.003	0.001	0.01		0

Institution Name	Overall Centrality	Biology Centrality	Biochemistry Centrality	Civil Engineering Centrality	Math Centrality
Rutgers, The State University of New Jersey - New Brunswick/Piscataway	0.003	0.001	0.002	0.001	0.007
Northern Arizona University	0.003	0.002	0.006		0.002
Michigan Technological University	0.003	0.001		0.011	0.001
Rice University	0.003	0	0.005	0.002	0.002
University of Rochester	0.003	0.002	0.004		0.006
Washington University in St Louis	0.003	0.006	0.003	0.001	0.001
Lehigh University	0.003	0.001	0.001	0.003	0.003
University of North Carolina at Greensboro	0.003	0.002	0.008		0
SUNY at Buffalo	0.003	0.002	0.001	0.006	0.001
University of Nevada - Reno	0.003	0.001	0.009	0.001	0
University of Utah	0.003	0.002	0.002	0.002	0.004
University of Illinois at Chicago	0.003	0.001	0	0.001	0.004
Illinois Institute of Technology	0.003	0.001		0.007	0.004
Oregon State University	0.003	0.003	0.004	0.004	0.004
Wake Forest University	0.003	0.004		0	0.004
New Jersey Institute of Technology	0.003			0.009	0.003
Auburn University Main Campus	0.003	0.003	0.003	0.003	0.001
North Carolina State University	0.003	0.002	0.001	0.01	0.001
Dartmouth College	0.003	0.003	0.005		0.003
South Dakota State University	0.003	0.001	0.005	0.004	0
Montana State University - Bozeman	0.003	0	0.007	0.003	0.005
University of Colorado Denver	0.003	0.001		0.006	0.004
Carnegie Mellon University	0.003	0	0.001	0.007	0.002
University of California - Santa Cruz	0.002	0.006	0.004		0.001
Vanderbilt University	0.002	0.001	0.001	0.005	0.002
University of Florida	0.002	0.003	0.001	0.004	0.002
Kansas State University	0.002	0.003	0	0.001	0.001
University of Houston	0.002	0.001	0.002	0.002	0.001
University of Hawaii at Manoa	0.002	0.005		0.001	
Florida Agricultural and Mechanical University	0.002			0.001	0.003
Jackson State University	0.002	0.002	0.001	0.003	0.002
University of North Carolina at Charlotte	0.002	0.002	0	0.003	0.004
Clarkson University	0.002	0.001		0.006	
Bryn Mawr College	0.002	0.004			0.002
University of Kentucky	0.002	0.002	0.001	0.004	0.001
University of Maryland - Baltimore County	0.002	0.001	0		0.002
University of Cincinnati - Main Campus	0.002	0.003	0	0.004	0.004
Utah State University	0.002	0.002	0.001	0.002	0.001

Institution Name	Overall Centrality	Biology Centrality	Biochemistry Centrality	Civil Engineering Centrality	Math Centrality
Temple University	0.002	0.002		0.001	0.002
University of Central Florida	0.002	0.001		0.01	0.001
George Washington University	0.002	0.005	0	0.002	0.002
University of Vermont	0.002	0.001	0.001	0.004	0.003
University of California - San Francisco	0.002	0.004	0.003		0.001
University of Oregon	0.002	0	0.003	0	0.004
Ohio University - Main Campus	0.002	0.001	0.002	0.001	0.001
North Carolina Central University	0.002	0.003	0.001	0	0.002
University of Cincinnati - Main Campus	0.002	0.003	0	0.004	0.004
University of New Hampshire-Main Campus	0.002	0.001	0	0.005	0.001
Washington State University	0.002	0.003	0.001	0.002	0
Clark University	0.002		0.005	0	0.002
Case Western Reserve University	0.002	0	0.003	0.002	0.001
University of Pittsburgh - Main Campus	0.002	0.001	0	0.005	0.001
Clemson University	0.002	0	0.004	0.005	0
University of Missouri - Columbia	0.002	0.004		0.004	0.003
University of Miami	0.002	0.004	0.001	0.001	
University of Akron	0.002	0.001		0.007	0
Tufts University	0.002	0.002	0.003	0	0.002
Southern Illinois University Carbondale	0.002	0.002	0	0.002	0.002
University of Louisiana at Lafayette	0.002	0.001		0.003	0.002
Bowling Green State University	0.002	0.002	0.001		0.003
Boston University	0.002	0.002	0.004	0.002	0.002
Marquette University	0.002	0.001		0.002	0.003
University of Nevada - Las Vegas	0.002	0		0.005	0.002
University of South Carolina - Columbia	0.002	0.001		0.003	0.002
Florida State University	0.002	0.003	0.001	0.001	0.004
University of Wyoming	0.002	0.001	0	0.002	0.004
University of Oklahoma	0.002	0.003	0.001	0.001	0.003
Rensselaer Polytechnic Institute	0.002	0	0.001	0.002	0.001
CUNY City College of New York	0.002	0.004		0.002	0.003
Wayne State University	0.002	0.002	0.001	0.001	0.001
University of Wisconsin - Milwaukee	0.001	0.001		0.001	0.001
New Mexico Institute of Mining and Technology	0.001	0.001		0.003	0
University of Missouri - St Louis	0.001	0.001	0		0
Medical College of Wisconsin	0.001	0.001			
College of William and Mary	0.001	0.003			0.001
Alabama A & M University	0.001	0.002		0.002	

Institution Name	Overall Centrality	Biology Centrality	Biochemistry Centrality	Civil Engineering Centrality	Math Centrality
University of Louisville	0.001	0		0.002	0
University of Denver	0.001	0	0.003		0
Saint Louis University-Main Campus	0.001	0		0	0.002
SUNY at Binghamton	0.001	0	0.001		0.002
Northeastern University	0.001	0.001		0	0.002
Indiana University of Pennsylvania	0.001	0.001			0.001
SUNY at Albany	0.001	0	0.001	0.001	0.002
Boston College	0.001	0.003	0.001		
Western Michigan University	0.001			0.001	0.005
Brandeis University	0.001	0.001	0.002		0.001
Kent State University	0.001	0.001			0.001
Georgia State University	0.001	0.001			0.002
Wright State University	0.001	0		0	0.002
Texas A & M University - Kingsville	0.001	0.002		0.002	0
Syracuse University	0.001	0.001		0.001	0.001
Illinois State University	0.001	0.001			0.003
Miami University	0.001	0.001			0.003
Colorado School of Mines	0.001		0.002	0.001	
Old Dominion University	0.001	0		0.001	0
University of Arkansas, Fayetteville	0.001	0.001	0.002		0
Florida Atlantic University	0.001	0.003	0.003	0	0.001
Oklahoma State University	0.001	0	0.003	0	0.002
University of the Incarnate Word	0.001	0.001			0.001
University of New Orleans	0.001	0	0.001	0	0.002
Virginia Commonwealth University	0.001	0.001	0.001		0
University of South Florida	0.001	0.002		0.003	0.001
University of Toledo	0.001		0	0	
University of Maine	0.001	0.002	0.002	0	0
SUNY College of Environmental Science and Forestry	0.001	0.003		0	
New York University	0.001	0.001	0	0.001	0.004
Wichita State University	0.001	0.001		0	0.001
University of Alabama in Huntsville	0.001	0.001		0.003	0
Hampton University	0.001	0.001			0.001
University of Texas Southwestern Medical Center at Dallas	0.001	0.001	0		
The University of Texas at Dallas	0.001	0.002			0
Mississippi State University	0.001	0.001		0.002	0.002
Texas Tech University	0.001	0.002		0	0

Institution Name	Overall Centrality	Biology Centrality	Biochemistry Centrality	Civil Engineering Centrality	Math Centrality
Tulane University	0.001	0.001	0	0	0.003
University of Mississippi Main Campus	0.001	0	0.001	0.002	0
Tennessee State University	0.001	0.001		0	0.002
Rockefeller University	0.001	0.002	0		
The University of Alabama	0.001	0.001	0.001	0.001	0.001
Emory University	0.001	0.004	0.002		0.001
Baylor College of Medicine	0.001	0.002			
Clark Atlanta University	0.001	0.001		0	0
University of North Texas	0.001	0.002			0
University of Northern Colorado	0.001	0	0		0.001
University of South Alabama	0.001	0		0.001	0.003
Florida Institute of Technology-Melbourne	0.001	0.002		0.004	0
Baylor University	0.001	0.001	0.002		0.001
University of Arkansas at Little Rock	0.001	0.001	0	0.001	0.001
Louisiana Tech University	0.001	0.001		0.002	
University of Massachusetts - Lowell	0.001	0	0	0.003	
University of Maryland - Eastern Shore	0.001	0.001		0.002	0.001
Stevens Institute of Technology	0.001			0.004	0
Georgetown University	0.001	0.001			0.001
Texas Woman's University	0.001	0.002			0
College of Medicine, Mayo Clinic	0		0.001		
Southern Methodist College	0				0
Claremont Graduate University	0				0.001
Teachers College at Columbia University	0	0			0
University of Southern Mississippi	0	0	0		
University of North Dakota	0	0.002			
American University	0		0		
Louisiana College	0				0
Thomas Jefferson University	0	0			
Muskingum University	0	0			
Arkansas State University	0	0.001			0
The University of Texas Health Science - San Antonio	0	0.001			0
CUNY Graduate School and University Center	0	0			0
Loma Linda University	0	0			
University of Medicine and Dentistry of New Jersey	0	0			
The University of Texas Medical Branch	0	0.001	0.001		
University of Rhode Island	0	0			

Institution Name	Overall Centrality	Biology Centrality	Biochemistry Centrality	Civil Engineering Centrality	Math Centrality
Fordham University	0	0			0.001
University of Massachusetts - Boston	0	0			0.001
Tennessee Technological University	0			0.002	
Meharry Medical College	0	0			
Nova Southeastern University	0				0
Texas A & M University System Health Science Center	0	0.001			
Texas Wesleyan University	0	0			
University of Memphis	0	0.001		0	0
University of Alabama at Birmingham	0	0.001	0.001	0.001	
The University of Texas Health Science Center at Houston	0	0.001	0		
Tuskegee University	0	0			0
Southern University and A & M College	0	0		0.002	0
Southern Methodist University	0	0		0	0
Cornell College	0		0.001		
University of Puerto Rico - Mayaguez	0			0.001	
Medical University of South Carolina	0	0			
Catholic University of America	0				0
McNeese State University	0	0		0.001	0
University of Mississippi Medical Center	0	0			
Yeshiva University	0				0.001
Oregon Health & Science University	0	0.001		0.001	
Texas State University - San Marcos	0				0
South Dakota School of Mines and Technology	0			0	

1. Clauset A, Arbesman S, Larremore DB. Systematic inequality and hierarchy in faculty hiring networks. Science advances. 2015;1: e1400005.

2. Flattau PE, Maher BA, Goldberger ML, others. Research Doctorate Programs in the United States:: Continuity and Change. National Academies Press; 1995.

3. US News and World Report. Top Graduate Programs [Internet]. [cited 30 Dec 2016]. Available: http://grad-schools.usnews.rankingsandreviews.com/best-graduate-schools

4. QS World University Rankings. In: Top Universities [Internet]. [cited 30 Dec 2016]. Available: http://www.topuniversities.com/qs-world-university-rankings

5. Baldi S. Prestige determinants of first academic job for new sociology Ph. Ds 1985–1992. The Sociological Quarterly. 1995;36: 777–789.
