# Supplementary material for: Take me where I want to go: Institutional prestige, advisor sponsorship, and academic career placement preferences
Source: PLoS One. 2017 May 11;12(5):e0176977. doi: 10.1371/journal.pone.0176977 (PMC5426638; doi:10.1371/journal.pone.0176977)
Supplement: S2 Appendix — (PDF) [file pone.0176977.s002.pdf]

Appendix S2: Scoring advisor support

Our measure of advisor involvement is based on a principal component factor analysis of responses to the following survey items: **During your first faculty job search, did your dissertation advisor do any of the following? (check all that apply)**

- Wrote recommendation letters
- Made phone calls on their behalf
- Defended career choice with others
- Gave advice on how to negotiate

Since the questions results in four binary variables, we use principal component factor analysis using polychoric correlations (PCA) [1], as is appropriate for combining discrete and binary variables into a measure that captures a concept as a single measure. The reason for doing so here, as opposed to simply adding them up as a single ordinal variable, is that doing PCA allows different factors to be weighted differently. If we were to measure advisor sponsorship as a single, ordinal variable we would violate the necessary assumption of equal spacing between the values (i.e., we would have to assume that the distance between an advisor who does not even write a letter of recommendation and one who only does that is the same as that between and advisor who writes a letter and an advisor who writes and calls). As a result, PCA allows us to measure a single dimension based on the 4 variables described above while taking into account the different factor loadings. The principal component estimated here explains 0.53, or about 53% of the variation in the 4 items. The scoring coefficient for each item can be seen in Table S2.1.

Table S2.1: Advisor Support Scoring Coefficients

	Scoring Coefficients
phone calls	
0	-0.13
1	0.73
recommendation letters	
0	-0.88
1	0.13
defend choices	
0	-0.11
1	0.89
gave advice on how to negotiate	
0	-0.21
1	0.58

The above procedure results in a normalized variable with mean 0 and standard deviation of 1. Our results are consistent if we treat the four items as simply an ordinal scale. As mentioned previously, recall bias may be a significant consideration for advisor related questions. Unfortunately, we do not have simpler questions of advisor sponsorship. Most other questions related to relationships with one's advisor relate to current relationship, and not relationship at time of PhD. Our results regarding differences in effects of advisor sponsorship along gender lines are consistent if we restrict our sample to just assistant and associate professors, or if we restrict our sample to only those with PhDs after 1995 (i.e., after the median year of completion), with the only difference being that the coefficient for research oriented males drops slightly below the 0.1 significance level obtained in the full sample.

1. Kolenikov S, Angeles G. Socioeconomic status measurement with discrete proxy variables: Is principal component analysis a reliable answer? Review of Income and Wealth. 2009;55: 128–165.
