# Supplementary material for: Take me where I want to go: Institutional prestige, advisor sponsorship, and academic career placement preferences
Source: PLoS One. 2017 May 11;12(5):e0176977. doi: 10.1371/journal.pone.0176977 (PMC5426638; doi:10.1371/journal.pone.0176977)
Supplement: S3 Appendix — (PDF) [file pone.0176977.s003.pdf]

S3: Relative Risk Ratio Tables

Within the main body of the paper, we present marginal effects. In a multinomial logit model, presenting information in this way makes interpretation of results substantially easier. However, for the sake of completeness, we also provide the results for relative risk ratios. These tables present the relative risk ratio of each alternative when compared to the baseline. These tables refer to the same models included in the paper, the only difference is that these use relative risk ratios in relation to the baseline instead of marginal effects.

Table S3.1: Relative Risk Ratios for Full Model

**Relative Risk Ratios - Research Extensive as Baseline**						
	**Research Intensive**		**Master's**		**Liberal Arts**	
	**Relative Risk Ratio**	**SE**	**Relative Risk Ratio**	**SE**	**Relative Risk Ratio**	**SE**
**Teaching Preference**	3.523***	0.832	13.578***	3.072	19.235***	4.167
**Advisor Sponsorship**	0.828*	0.09	0.833*	0.082	0.843	0.1
**Advisor Sponsorship*Teaching Preference**	1.267	0.273	1.225	0.237	1.251	0.255
**Doctoral Prestige**	0.650***	0.064	0.630***	0.071	0.864	0.088
**Doctoral Prestige*Teaching preference**	1.405	0.342	1.398	0.342	1.557*	0.355
**Best dissertation award received**	0.364***	0.137	0.434***	0.123	0.423***	0.134
**Year PhD completed**	1.022***	0.008	1.044***	0.008	1.016**	0.008
**Biochemistry**	0.630**	0.137	0.603**	0.127	3.188***	0.637
**Civil engineering**	0.634**	0.135	0.553***	0.125	0.336***	0.094
**Math**	1.318	0.297	1.581**	0.344	1.965***	0.44
**Dependent child at PhD**	1.129	0.232	1.117	0.227	1.081	0.224
**Female**	0.936	0.16	0.688**	0.121	1.3	0.234
**First Generation College Graduate**	0.887	0.166	0.843	0.155	0.387***	0.076
**African American**	1.032	0.408	1.769	0.631	1.698	0.674
**Hispanic**	0.432*	0.201	1.069	0.335	1.226	0.439
**Native American/Alaskan**	3.119	2.817	1.76	1.34	1.054	0.897
**Asian**	1.13	0.225	1.419*	0.264	0.962	0.219
**Other Race/Ethnicity**	0.179	0.301	0.26	0.504	0.426	0.524
**Constant**	0.000***	0	0.000***	0	0.000**	0

*** p<0.1, ** p<0.05, ***p<0.01**

Table S3.2: Relative Risk Ratios for Male only model

Relative Risk Ratios - Research Extensive as Baseline						
	Research Intensive		Masters		Liberal Arts	
Teaching Preference	3.144***	1.078	14.308***	4.567	20.683***	6.387
Advisor Sponsorship	0.807	0.116	0.863	0.106	0.762*	0.125
Advisor Sponsorship*Teaching Preference	1.279	0.401	1.016	0.28	1.207	0.357
Doctoral Prestige	0.660***	0.081	0.657***	0.092	0.837	0.11
Doctoral Prestige*Teaching preference	1.291	0.446	1.277	0.435	1.398	0.451
Best dissertation award received	0.361**	0.16	0.372***	0.127	0.451**	0.18
Year PhD Completed	1.026**	0.01	1.056***	0.01	1.028***	0.01
Biochemistry	0.543**	0.153	0.505**	0.141	3.146***	0.893
Civil engineering	0.593*	0.16	0.529**	0.152	0.338***	0.127
Mathematics	1.166	0.346	1.341	0.388	2.161**	0.672
Dependent child at PhD	1.221	0.315	1.368	0.341	1.292	0.343
Female	1	(.)	1	(.)	1	(.)
First Generation College Graduate	0.914	0.214	0.865	0.197	0.391***	0.097
African American	0.823	0.393	1.286	0.562	1.999	0.953
Hispanic	0.577	0.351	0.932	0.409	1.815	0.887
Native American/Alaskan	3.607	3.755	1.747	1.575	0.000***	0
Asian	1.05	0.266	1.267	0.299	0.759	0.243
Other Race/Ethnicity	0.033**	0.054	0.014***	0.022	0.136*	0.159
Constant	0.000***	0	0.000***	0	0.000***	0

*** p<0.1, ** p<0.05, ***p<0.01**

Table S3.3: Relative Risk Ratios for Female Only Model

Relative Risk Ratios - Research Extensive as Baseline						
	Research Intensive		Masters		Liberal Arts	
Teaching Preference	5.077***	1.378	16.598***	4.312	22.211***	6.121
Advisor Sponsorship	0.89	0.125	0.805	0.124	1.028	0.159
Advisor Sponsorship*Teaching Preference	1.479*	0.345	2.054***	0.457	1.508*	0.35
Doctoral Prestige	0.665***	0.093	0.584***	0.093	0.969	0.152
Doctoral Prestige*Teaching Preference	1.522	0.437	1.555	0.44	1.572*	0.426
Best dissertation award received	0.295**	0.162	0.624	0.255	0.337**	0.174
Year PhD Completed	1.012	0.012	1.012	0.012	0.992	0.011
Biochemistry	0.86	0.268	0.824	0.252	3.731***	1.039
Civil engineering	0.675	0.216	0.414**	0.157	0.329***	0.137
Mathematics	1.766**	0.458	2.146***	0.541	2.000**	0.545
Dependent child at PhD	0.984	0.295	0.79	0.252	0.806	0.259
Female	1	(.)	1	(.)	1	(.)
First Generation College Graduate	0.979	0.272	1.01	0.287	0.456**	0.139
African American	1.878	0.886	3.885***	1.574	2.339	1.222
Hispanic	0.254**	0.151	1.082	0.461	0.495	0.286
Native American/Alaskan	1.772	2.346	2.104	2.142	3.894	3.598
Asian	1.361	0.393	1.779**	0.495	1.32	0.426
Other Race/Ethnicity	2.778	3.314	12.816***	9.934	4.988*	4.128
Constant	0	0	0	0	1.76E+06	3.95E+07

*** p<0.1, ** p<0.05, ***p<0.01**
